# Maternal Immune Response to ZIKV Triggers High-Inflammatory Profile in Congenital Zika Syndrome

**DOI:** 10.3390/v15010220

**Published:** 2023-01-13

**Authors:** Eder M. S. Fialho, Emanoel M. Veras, Caroline M. de Jesus, Ricardo Khouri, Patrícia S. Sousa, Marizelia R. C. Ribeiro, Luciana C. Costa, Líllian N. Gomes, Flávia R. F. Nascimento, Antônio A. M. Silva, Paulo V. Soeiro-Pereira

**Affiliations:** 1 Health Sciences Graduate Program, Federal University of Maranhão, São Luís 65080-805, MA, Brazil; 2 Medical School, Federal University of Maranhão, São Luís 65080-805, MA, Brazil; 3 Health and Technology Graduate Program, Federal University of Maranhão, São Luís 65080-805, MA, Brazil; 4 Gonçalo Moniz Research Institute, FIOCRUZ-Bahia, Salvador 40296-710, BA, Brazil; 5 Reference Center on Neurodevelopment, Assistance and Rehabilitation of Children/NINAR–State Department of Health of the State of Maranhão, São Luís 65077-357, MA, Brazil; 6 Department of Medicine III, Federal University of Maranhão, São Luís 65020-240, MA, Brazil; 7 Department of Public Health, Federal University of Maranhão, São Luís 65020-060, MA, Brazil; 8 Department of Immunology, University of São Paulo, São Paulo 05508-000, SP, Brazil; 9 Department of Pathology, Federal University of Maranhão, São Luís 65065-545, MA, Brazil

**Keywords:** reactive oxygen species, microcephaly, monocytes, Zika virus infection

## Abstract

The immunological mechanisms involved in the development of congenital Zika syndrome (CZS) have yet to be fully clarified. This study aims to assess the immuno-inflammatory profile of mothers and their children who have been diagnosed with CZS. Blood samples, which were confirmed clinically using the plaque reduction neutralization test (PRNT), were collected from children with CZS and their mothers (CZS+ group). Samples were also collected from children who did not develop CZS and had a negative PRNT result and from their mothers (CZS- group). The data demonstrated a correlation between the leukocyte profile of CZS+ children and their mothers, more evident in monocytes. Monocytes from mothers of CZS+ children showed low expression of HLA and elevated hydrogen peroxide production. CZS+ children presented standard HLA expression and a higher hydrogen peroxide concentration than CZS- children. Monocyte superoxide dismutase activity remained functional. Moreover, when assessing the monocyte polarization, it was observed that there was no difference in nitrite concentrations; however, there was a decrease in arginase activity in CZS+ children. These data suggest that ZIKV infection induces a maternal immuno-inflammatory background related to the child’s inflammatory response after birth, possibly affecting the development and progression of congenital Zika syndrome.

## 1. Introduction

At the end of 2015, Brazil registered an outbreak of childbirths with a set of manifestations associated with the infection of mothers by Zika virus (ZIKV), congenital Zika syndrome (CZS) [1,2,3]. Clinical features included microcephaly, ventriculomegaly, calcifications, arthrogryposis, and cutis gyrata [4]. These manifestations are associated with the neurotropism of the virus, which leads to a more severe spectrum of abnormalities in the central nervous system [5]. ZIKV was replicated successfully in an in vitro model (U87-MG neural cells lineage), causing inflammation and cell death [6].

Our study group has already described the changes caused by ZIKV infection during pregnancy in a group of children, observing, intriguingly, that the children showed an increase in their head circumference Z-score after 24 months, indicating an ongoing inflammatory response during this period [7,8].

The inflammation induced by the infection appears to be a critical viral mechanism for nerve-cell degeneration [9]. Tissue damage, initially associated with virus-induced apoptosis, may also be related to the immune response and not the virus itself [10]. Various studies demonstrate that leukocytes are activated during the antiviral response to eliminate the pathogen but can produce mediators of damage (e.g., tumor necrosis factor-alpha, interleukins-1, 6, and 17) [11,12].

Reactive oxygen species (ROS) are essential mediators in the increased damage caused by the inflammatory response [13]. Among the primary ROS-producing cells are neutrophils and monocytes/macrophages, as these are efficient phagocytes that eliminate microorganisms at the site of infection. Within the inflammatory environment, macrophages are the first cells to interact with viral pathogens in loco, directing the immune response through their M1 (pro-inflammatory) and M2 (regulatory) polarization [14]. On the other hand, neutrophils are little related to the antiviral response. Still, their release of antimicrobial agents and their NETs are essential mechanisms for controlling intracellular infections [15].

Thus, we believe that the participation of both neutrophils and macrophages in ROS production during the inflammatory response may contribute to the progression of CZS. The change in the redox status of patients infected with Dengue virus, demonstrated by the increase in serum protein and lipid-oxidation markers, is related to an increase in inflammatory response, cell death, and disease severity [16].

Initial oxidative stress contributes to increased infection in monocytes [17]. Thus, ROS can assume an ambiguous role during infection, exerting a modulation in different cellular redox stress pathways that increases the damage caused by the inflammatory response [18]. However, they are also essential components of the host response against viral infection [19].

Few studies have demonstrated the mechanisms of the immunopathogenesis of ZIKV infection, especially in mothers and their children with confirmed CZS [20,21]. The present study shows an inflammatory profile in children with CZS and their mothers, suggesting that ZIKV infection induces a maternal immuno-inflammatory response with an increased monocyte oxidative response. Thus, such inflammatory mediators could be sources of investigation for possible markers in the context of ZIKV infection in mothers during pregnancy and their children with CZS.

## 2. Material and Methods

### 2.1. Ethical Aspects

The study complied with all the definitions of the Declaration of Helsinki. It was submitted to and approved by the Research Ethics Committee of the Federal University of Maranhão Hospital (CAAE: 65897317.1.0000.5086), with all procedures performed only after the reading and signing of the informed consent form.

### 2.2. Study Design

The study included a prospective cohort of children living in the State of Maranhão in northeastern Brazil and was conducted jointly with the Reference Center for Neurodevelopment, Assistance, and Rehabilitation of Children (NINAR).

The prospective cohort included children with at least one of the following abnormalities in the cranial imaging examination: calcifications, reduced cerebral parenchyma volume, ventriculomegaly, cortical developmental malformation, cerebellar malformation/hypoplasia, trunk malformation/hypoplasia, and agenesis/dysgenesis of the corpus callosum [8]. The study excluded children with other congenital syndromes (e.g., positive TORCHS).

Clinical data were collected using a structured questionnaire. Blood (5 mL) was drawn from the mother and child using an EDTA tube. The children’s anthropometric data (length, weight, and head circumference) were normalized using the standard curve of INTERGROWTH-21st (International Fetal and Neonatal Growth Consortium for the 21st Century), which considers gestational age and sex. This study defines microcephaly as a head-circumference measurement of less than two standard deviations (SDs) below the average [8].

Of 166 children referred to NINAR for investigation of CZS, 103 underwent PRNT50 from July 2017 to July 2018. Of the 63 who had a negative PRNT results, 30 children, constituting the control group (CZS–), presented no signs or symptoms of CZS. From a total of 40 children with CZS confirmed by PRNT (CZS+), blood samples for this study were drawn from 27 children. There were no adequate tests or proper examinations at the beginning of the pandemic. A few children and their mothers underwent RT-PCR (with undetectable viral load or indeterminate result) or IgM antibody dosage for Zika and Dengue (twelve positives, eight indeterminates, and seven negatives). Thus, the PRNT was essential to characterize the populations studied in terms of exposure to the virus and consequent maintenance of the immune/inflammatory response.

### 2.3. PRNT (Plaque Reduction Neutralization Test)

The PRNT was performed on the basis of the protocol previously described [22], with some modifications. The cutoff value for PRNT positivity was 50% (PRNT50), which means the maximum serum dilution (1:8 to 1:4096) needed to reduce the formation of ZIKV plaque by 50% [23]. We used a Vero cell (2 × 105 cell/well) and the ZIKV PE/243 strain (100 PFU/well) isolated in Brazil. All sera were inactivated (56 °C, 60 min) before the neutralization test. Serum samples were serially diluted in Dulbecco’s Modified Eagle’s medium (DMEM, Gibco, New York, NY, USA) with 2% fetal bovine serum (FBS, Gibco, NEW York, NY, USA) and 1% penicillin/streptomycin. Then, 250 µL of virus suspension (100 PFU/well) was added to each well containing diluted serum (1: 1). Dilutions of serum and virus were then incubated at 37° C for 60 min. A final volume of 200 µL of each serum dilution and virus mix was transferred to a plate containing Vero cells and then incubated at 37 °C for 60 min. After incubation, 300 µL of 0.6% agarose solution was added, and the plates were re-incubated at 37 °C for five days. The reactions were then revealed using a 2% Naphthol Blue Black solution (Sigma-Aldrich, St. Louis, MO, USA). PRNT 50 ≥ 10 was considered positive.

### 2.4. Immunophenotyping

Leukocytes from hemolyzed peripheral blood samples (100 µL) were incubated with antibodies conjugated to fluorochromes at 4 °C for 30 min. Then, the cells were washed with a phosphate-buffered solution for data acquisition in a BD FACSCalibur flow cytometer (BD Biosciences, San Diego, CA, USA).

Monocyte and neutrophil populations were characterized by CD14/HLA-DR and CD15/CD66b/MPO (Becton, Dickinson, and Company, Franklin Lakes, NJ, USA), respectively. Data analysis was performed using FlowJo software (Becton, Dickinson and Company, Franklin Lakes, NJ, USA). The results are presented in the percentage of cells expressing the markers and their expression levels, indirectly determined by the mean fluorescence intensity (MFI).

### 2.5. Hydrogen Peroxide Production

This analysis was performed by indirect quantification of the production of hydrogen peroxide (H_2_O_2_) by the oxidation of dihydrorhodamine (DHR)-123 to rhodamine-123 (Sigma-Aldrich, St. Louis, MO, USA) [24]. Briefly, peripheral blood monocytes and neutrophils were stimulated with PMA (Phorbol 12-myristate 13-acetate, 50 nM) (Sigma-Aldrich, St. Louis, MO, USA) or only medium for one hour. Then, DHR (200 ng/mL) was added to the cells, which were incubated for 30 min for later data acquisition in a BD FACSCalibur flow cytometer. The analysis was performed using FlowJo software (Becton, Dickinson, and Company, Franklin Lakes, NJ, USA). We obtained data on the percentage of H_2_O_2_-producing cells and the amount of this reactive, as determined by the MFI values.

### 2.6. Superoxide Dismutase (SOD)

The SOD Assay Kit (Sigma-Aldrich, St. Louis, MO, USA) was used according to the manufacturer’s instructions. Briefly, after incubation at 37 °C for 20 min with xanthine oxidase solution and tetrazolium salt, the samples were read on a spectrophotometer using an absorbance of 450 nm. The results were expressed as SOD activity (%).

### 2.7. Arginase Activity

The Arginase Activity Assay Kit (Sigma-Aldrich, St. Louis, MO, USA) was used according to the manufacturer’s instructions. Physiological urea was removed from the samples and incubated at 37 °C for 2 h with a buffered substrate containing arginine. The reaction was stopped by adding urea reagent. The absorbances were read on a spectrophotometer at 430 nm. The results were expressed in units/liter (one unit of arginase = amount of enzyme that converts one µmole of L-arginine to ornithine and urea per minute).

### 2.8. Nitric Oxide Evaluation

The evaluation of nitric oxide production was carried out indirectly through adaptation in the methodology for the measurement of nitrate [25]. In the present study, the procedure consisted, initially, of plasma deproteinization followed by the addition of 0.08% vanadium chloride solution. The reaction was revealed by adding the Griess reagent. The absorbances were read on a spectrophotometer at 540 nm. After subtracting the values from their respective blanks, a standard curve determined the nitrate concentration, and the results were expressed in µmol/L.

### 2.9. Statistical Analysis

The Shapiro–Wilk test assessed the normality of the data. According to this, Student’s t-test and the Mann–Whitney test were used. The data were expressed as mean ± standard deviation, and the differences were considered significant when *p* < 0.05. Pearson’s correlation coefficient verified the association between continuous variables. GraphPad Prism v.7 software was used for these analyses.

## 3. Results

### 3.1. Immunophenotypic Profile of CZS+ Children and Their Mothers Are Similar

**Figure 1:**
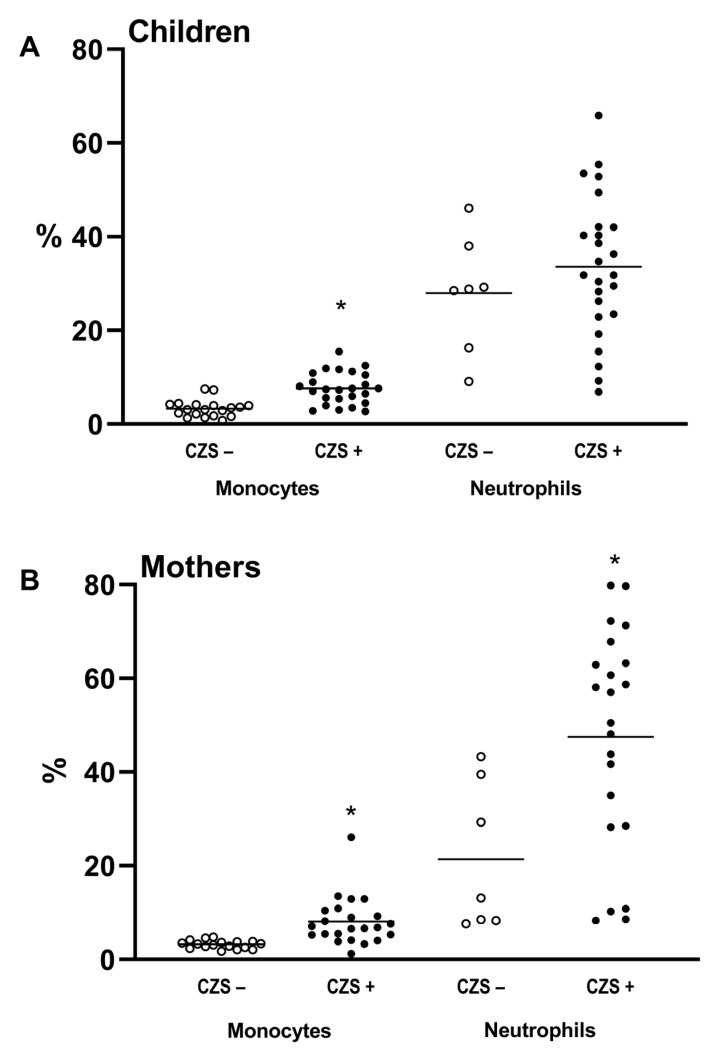
Immunophenotypic profile of CZS+ children and their mothers. Peripheral blood was collected from mothers and children, and the percentage of monocytes and neutrophils was quantified. The CZS– group (N = 30), represented by the hollow circles, and the CZS+ group (N = 27), represented by the filled circles, were compared as between children (**A**) and mothers (**B**). Each point represents an individual, and the bar represents the group’s median. Student’s t-test was used to compare the groups; (*) *p* < 0.05.

Immunophenotyping characterized CD14+ monocyte and CD15/CD66b+ neutrophil populations in CZS development. The cellular immune profiles of children without CZS and those with CZS were compared, as were those of their mothers. The comparison between CZS– and CZS+ children showed no differences in the percentage of neutrophils (Figure 1A; *p* = 0.3789). However, there was an increase in the percentage of monocytes in CZS+ compared to CZS– children (Figure 1A; *p* < 0.0001). Regarding mothers, the CZS+ group showed an increased percentage of neutrophils (Figure 1B; *p* < 0.0103) and monocytes (Figure 1B; *p* < 0.0001) compared to mothers of CZS– children.

### 3.2. Characterization of the CZS+ Neutrophil Population

**Figure 2:**
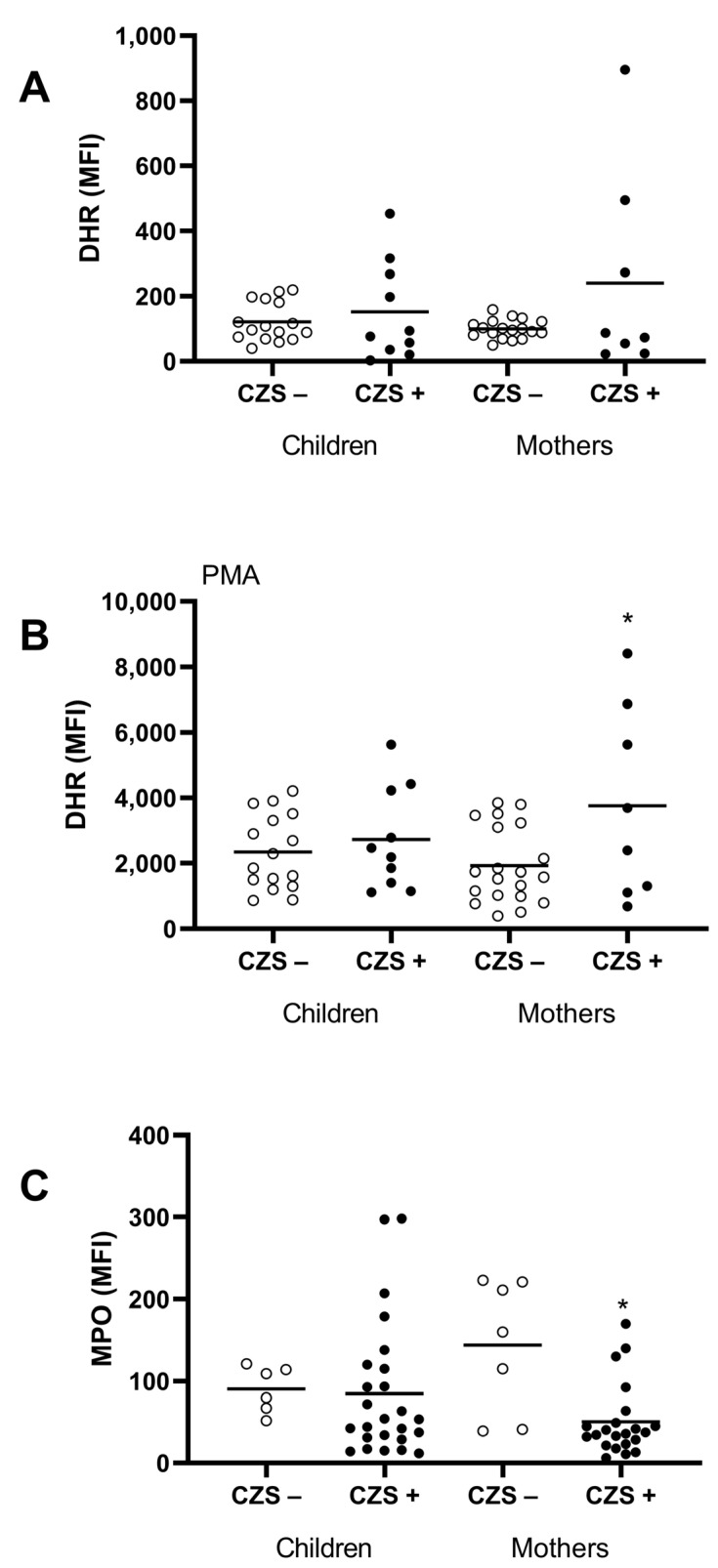
Neutrophil markers of oxidative stress (hydrogen peroxide and myeloperoxidase) of CZS+ children and their mothers. Peripheral blood was collected from CZS– (N = 30) and CZS+ (N = 27) children and their mothers. The cells were used to evaluate spontaneous (**A**) and PMA-stimulated (**B**) hydrogen peroxide production by monocytes (**A**) and neutrophils (**B**) using DHR assay and MPO expression intracell using flow cytometry (**C**). Each point represents an individual, and the bar represents the group’s median. Student’s t-test was used to compare the groups; (*) *p* < 0.05.

Neutrophils are essential ROS-producing cells, and hydrogen peroxide is one of the main molecules. Thus, we evaluated hydrogen peroxide production using DHR assay and we verified the expression of the myeloperoxidase (MPO) enzyme involved in H_2_O_2_ metabolization. The CZS+ children showed no differences in hydrogen peroxide production, measured by the DHR test, or concerning the expression of the MPO enzyme, verified using flow cytometry, in the neutrophil population. However, CZS+ mothers showed increased hydrogen peroxide production by neutrophils when stimulated with PMA (*p* = 0.0221). We also found that CZS+ mothers presented a reduced neutrophil MPO expression (Figure 2; *p* = 0.0005).

### 3.3. Characterization of the CZS+ Monocyte Population

**Figure 3:**
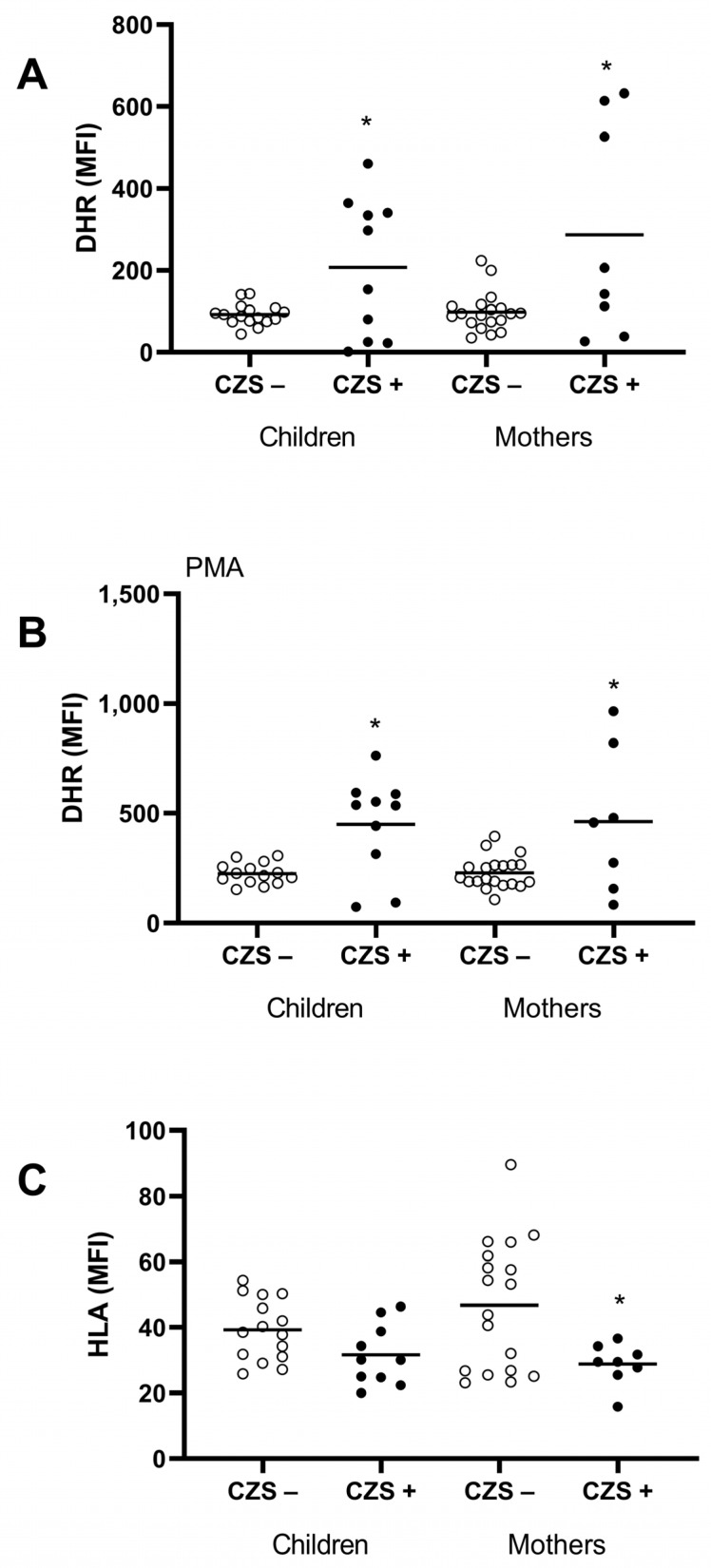
Monocyte markers of oxidative stress (hydrogen peroxide and superoxide dismutase) of CZS+ children and their mothers. Peripheral blood was collected from CZS– (N = 30) and CZS+ (N = 27) children and their mothers. The cells were used to evaluate spontaneous (**A**) and PMA-stimulated (**B**) hydrogen peroxide production through DHR assay and the HLA expression on the surface via flow cytometry (**C**). Each point represents an individual, and the bar represents the group’s median. Student’s t-test was used to compare the groups; (*) *p* < 0.05.

Monocytes are ROS-producing cells and are also a fundamental bridge between innate and adaptive immune response by antigen presentation through the HLA surface molecule. One essential feature of activated monocytes is the production of reactive oxygen species. Both the CZS+ children (*p* = 0.0014) and their mothers (*p* = 0.0051) showed an increase in hydrogen peroxide production (Figure 3A,B). There was no difference in HLA expression between CZS- and CZS+ children (Figure 3C; *p* = 0.0560). However, monocytes from mothers of CSZ+ children presented a lower expression of HLA molecules than monocytes from mothers of CSZ- children (Figure 3C; *p* = 0.0206).

**Figure 4:**
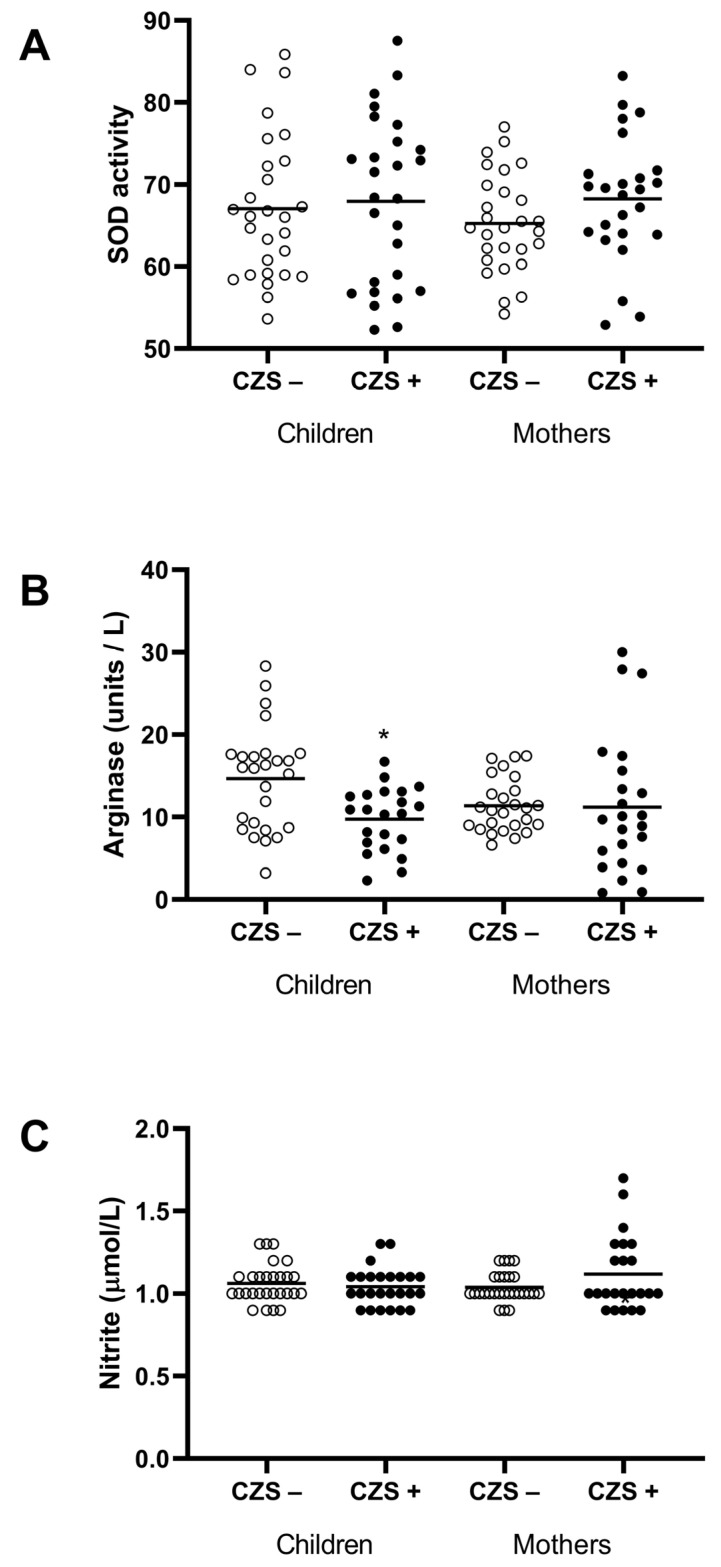
Markers of polarization (SOD, arginase, and nitrites) of CZS+ children and their mothers. The plasma was used to evaluate the SOD activity (**A**), the arginase activity (**B**), and the nitric oxide production (**C**). Each point represents an individual, and the bar represents the group’s median. Student’s t-test was used to compare the groups; (*) *p* < 0.05.

This increase does not seem to be correlated with antioxidant enzymes, since SOD was not altered in those children (*p* = 0.7342) and their mothers (*p* = 0.1159) (Figure 4A).

### 3.4. Decreased M2 Response in Monocytes from CZS+ Children

Due to the high production of free radicals, we investigated monocyte polarization (M1 or M2) through plasmatic markers, such as nitrite and arginase enzymes. There were no differences in plasma nitrite concentration (indirect nitric oxide measurement) between CZS+ and CZS- children and their respective mothers (Figure 4C). On the other hand, arginase activity, a characteristic enzyme of alternative activation of macrophages, was reduced in CZS+ children (Figure 4B; *p* = 0.0206), reinforcing the idea of a systemic M1 inflammatory profile.

## 4. Discussion

Since the Brazilian microcephaly outbreak that occurred between 2015 and 2016, many studies have already demonstrated the association between Zika virus infection during pregnancy and congenital malformations [26,27]. However, few studies have produced data on the immunological characteristics of children with CZS and their mothers. In our work, we have shown an increase in the redox activity of children associated with the same pattern in their mothers. These results are in-human evidence that the maternal pro-inflammatory profile is possibly related to the development of CZS in children, which maintains a similar response profile.

Cugola et al. [28] showed that the Th1 immune response pattern is relevant for the resolution of ZIKV infection in its early stages and for fetus safety. Thus, the inflammatory cellular immune response plays a protective role during the infection. However, the inflammatory response could be harmful without regulatory mechanisms or if presented in an exacerbated and continuous manner [29]. Microcephaly is likely to be, at least in part, the result of an inflammatory process related to the mother’s viral infection, and this response can persist in the child even after birth [30,31].

The inflammatory response, its initiation, and its deleterious potential are intrinsically related to the number and function of phagocytes. The mothers in our study showed an increase in CD14 monocytes, as did their children. An in vivo and ex vivo study has demonstrated that CD14 monocytes from healthy pregnant women are susceptible to Zika virus infection [14]. This condition may favor the infection’s immunopathology by providing a more significant replication site and viral persistence. Many studies have linked this cell with viral replication and survival sites due to its plastic characteristics in tissues and cell–cell contact-dependent functions [32].

Another relevant aspect of phagocyte response in this inflammatory context is reactive oxygen species (ROS) production [33,34]. Oxidative stress influences the progression of viral infection in monocytes [35,36]. Despite the limited number of children in the analysis of the redox response, we can observe that CZS+ children who had monocytes with a more outstanding production of free radicals are the children of mothers with the same profile. Few studies have investigated the role of the redox response in the immunopathology of Zika virus infection. To date, our study is the first to provide evidence of the function of oxidative stress in congenital Zika syndrome.

Frequently, ROS production is related to damage to the cell structure due to its high reactivity [37]. Indeed, the severity of arboviruses during the acute phase is associated with high ROS production [38,39]. However, the increase in the oxidative response may be related to the faster replication and increased release of viral particles. Bhaskar et al. (2015) observed that an increased antioxidant response leads to resistance to apoptosis and viral latency in monocytes infected with the human immunodeficiency virus (HIV). In contrast, a decrease in this response leads to the reactivation of viral replication and the continuation of infection [40].

So far, we have examined a population of monocytes with a high production of free radicals, possibly associated with their clinical condition. ROS are necessary cellular signals in cell differentiation, proliferation, and death when adequately controlled [41]. However, altering the redox state by the accumulation of ROS can lead to the development of embryonic pathologies, such as abortions and intrauterine growth restriction [42].

One mechanism that may explain the lack of immune regulation is the decrease in HLA expression in maternal monocytes. There is still no described association between this molecule and CZS, nor even the Zika virus infection itself. However, HLA structure and expression variations can compromise the antigen presentation, leading to susceptibility to viral infections [43]. Our results suggest that mothers of children with CZS presented low regulatory mechanisms to control the inflammation, which can lead to harmful effects on the fetus.

Besides the production of free radicals, our results show that this response is associated with an M1 pro-inflammatory monocyte profile. Children’s monocyte presented a decrease in arginase activity, which occurs with a higher activity of nitric oxide synthetase (iNOS) [44]. However, these results conflict with Azevedo et al. (2018). They demonstrated that neural tissue samples from fatal CZS cases showed an inflammatory infiltrate with necrosis, apoptosis, and prevalence of M2 macrophages [20]. This difference is probably related to different site responses, one in the peripheral blood and the other in the tissue. Depending on the stage of the inflammatory process, the tissue macrophage tends to have a more resolute profile [45]. Unlike the children, the mothers did not show a decreased arginase activity. However, they also did not have any ongoing inflammatory process as in the case of congenital syndrome in the children and, therefore, did not show a more evident polarization pattern.

The evaluation of plasma nitrite concentration (indirect method of measuring nitric oxide) showed no difference between groups of children nor between their respective mothers. This radical’s short half-life justifies the similar NO-production pattern. Another explanation could be the reaction of NO with a variety of molecules, leading to the production of peroxynitrites (ONOO^−^), dinitrogen trioxide (N_2_O_3_), or nitronium ion (NO_2_^+^) [46]. This difference was possibly more evident during pregnancy, since, despite a suppressed maternal immune response, the presence of the Zika virus in the placenta causes a chronic inflammatory response, as already demonstrated by other studies [47]. Perhaps, for this reason, the maternal–fetal immune response polarizes towards a more inflammatory profile, with increased hydrogen peroxide production, as demonstrated in our study.

However, although we found increased levels of hydrogen peroxide in cells, SOD activity did not differ between CZS+ children or their mothers. During Dengue virus infection, the enzyme complex NADPH oxidase is activated. This complex is an essential producer of superoxide anion [19], which is reduced to hydrogen peroxide by the SOD enzyme [48]. Our results suggest the involvement of other oxidative pathways in the increase in hydrogen peroxide levels. Moreover, enzymes such as glutathione and catalase are related to the increased production of this molecule during viral infections, a crucial object of study for future investigations [41,49].

Together, these results reinforce the idea of an inflammatory profile, which can lead to a persistent inflammatory response. Some researchers show the association of redox imbalance with the course of infection by flaviviruses, such as hepatitis C and Dengue virus [50,51]. However, there are no studies that relate the redox response in CZS. In addition, changes in the mechanisms involved in the cellular redox balance can lead to inadequate cell signals, increasing deleterious immune effects [52].

## 5. Conclusions

Thus, our results show that the maternal pro-inflammatory immune profile probably triggers the development and progression of congenital Zika syndrome. The fact that individuals with CZS present an increase in oxidative stimuli may predispose them to more severe sequelae due to the damage caused by the excess of free radicals. The presence of cellular and soluble markers of persistent inflammation, even months after birth, indicates that the Zika virus and maternal immune response affect the development of children’s immune systems. Thus, the immunological analysis of these patients must be accompanied by clinical evaluation to make an adequate prognostic model for CZS.

## Data Availability

The data are part of a larger project and are still under bioinformatics analyses and correlation tests. As such, they are not yet publicly available. However, we make ourselves available to resolve any doubts regarding obtaining the results of this article.

## References

[1] Gorshkov K., Shiryaev S.A., Fertel S., Lin Y.W., Huang C.T., Pinto A., Farhy C., Strongin A.Y., Zheng W., Terskikh A.V. (2018). Zika Virus: Origins, Pathological Action, and Treatment Strategies. Front. Microbiol..

[2] Baud D., Gubler D.J., Schaub B., Lanteri M.C., Musso D. (2017). An Update on Zika Virus Infection. Lancet.

[3] Ribeiro L.S., Marques R.E., De Jesus A.M.R., De Almeida R.P., Teixeira M.M. (2016). Zika Crisis in Brazil: Challenges in Research and Development. Curr. Opin. Virol..

[4] van der Linden V., Pessoa A., Dobyns W., Barkovich A.J., van der Linden Júnior H., Filho E.L.R., Ribeiro E.M., de Carvalho Leal M., de Araújo Coimbra P.P., de Fátima Viana Vasco Aragão M. (2016). Description of 13 Infants Born During October 2015–January 2016 With Congenital Zika Virus Infection Without Microcephaly at Birth—Brazil. MMWR. Morb. Mortal. Wkly. Rep..

[5] Levine D., Jani J.C., Castro-Aragon I., Cannie M. (2017). How Does Imaging of Congenital Zika Compare with Imaging of Other TORCH Infections?. Radiology.

[6] Tricarico P.M., Caracciolo I., Crovella S., D’Agaro P. (2017). Zika Virus Induces Inflammasome Activation in the Glial Cell Line U87-MG. Biochem. Biophys. Res. Commun..

[7] Ribeiro M.R.C., Khouri R., Sousa P.S., Branco M.R.F.C., Batista R.F.L., Costa E.P.F., Alves M.T.S.S.B., Amaral G.A., Borges M.C.R., Takahasi E.H.M. (2020). Plaque Reduction Neutralization Test (PRNT) in the Congenital Zika Syndrome: Positivity and Associations with Laboratory, Clinical, and Imaging Characteristics. Viruses.

[8] Silva A.A.M., Ganz J.S.S., da Silva Sousa P., Doriqui M.J.R., Ribeiro M.R.C., dos Remédios Freitas Carvalho Branco M., de Sousa Queiroz R.C., de Jesus Torres Pacheco M., Vieira da Costa F.R., de Sousa Silva F. (2016). Early Growth and Neurologic Outcomes of Infants with Probable Congenital Zika Virus Syndrome. Emerg. Infect. Dis..

[9] Li H., Saucedo-Cuevas L., Regla-Nava J.A., Chai G., Sheets N., Tang W., Terskikh A.V., Shresta S., Gleeson J.G. (2016). Zika Virus Infects Neural Progenitors in the Adult Mouse Brain and Alters Proliferation. Cell Stem Cell.

[10] Lum F.M., Low D.K.S., Fan Y., Tan J.J.L., Lee B., Chan J.K.Y., Rénia L., Ginhoux F., Ng L.F.P. (2017). Zika Virus Infects Human Fetal Brain Microglia and Induces Inflammation. Clin. Infect. Dis..

[11] Lum F.-M., Lee D., Chua T.-K., Tan J.J.L., Lee C.Y.P., Liu X., Fang Y., Lee B., Yee W.-X., Rickett N.Y. (2018). Zika Virus Infection Preferentially Counterbalances Human Peripheral Monocyte and/or NK Cell Activity. mSphere.

[12] Yockey L.J., Iwasaki A. (2018). Role of Interferons and Cytokines in Pregnancy and Fetal Development. Immunity.

[13] Camini F.C., da Silva Caetano C.C., Almeida L.T., de Brito Magalhães C.L. (2017). Implications of Oxidative Stress on Viral Pathogenesis. Arch. Virol..

[14] Foo S.S., Chen W., Chan Y., Bowman J.W., Chang L.C., Choi Y., Yoo J.S., Ge J., Cheng G., Bonnin A. (2017). Asian Zika Virus Strains Target CD14+blood Monocytes and Induce M2-Skewed Immunosuppression during Pregnancy. Nat. Microbiol..

[15] Naumenko V., Turk M., Jenne C.N., Kim S.-J. (2018). Neutrophils in Viral Infection. Cell Tissue Res..

[16] Gil L., Martínez G., Tápanes R., Castro O., González D., Bernardo L., Vázquez S., Kourí G., Guzmán M.G. (2004). Oxidative Stress in Adult Dengue Patients. Am. J. Trop. Med. Hyg..

[17] Al-alimi A.A., Ali S.A., Al-Hassan F.M., Idris F.M., Teow S.Y., Mohd Yusoff N. (2014). Dengue Virus Type 2 (DENV2)-Induced Oxidative Responses in Monocytes from Glucose-6-Phosphate Dehydrogenase (G6PD)-Deficient and G6PD Normal Subjects. PLoS Negl. Trop. Dis..

[18] Valadão A.L.C., Aguiar R.S., de Arruda L.B. (2016). Interplay between Inflammation and Cellular Stress Triggered by Flaviviridae Viruses. Front. Microbiol..

[19] Olagnier D., Peri S., Steel C., van Montfoort N., Chiang C., Beljanski V., Slifker M., He Z., Nichols C.N., Lin R. (2014). Cellular Oxidative Stress Response Controls the Antiviral and Apoptotic Programs in Dengue Virus-Infected Dendritic Cells. PLoS Pathog..

[20] Azevedo R.S.S., de Sousa J.R., Araujo M.T.F., Martins Filho A.J., de Alcantara B.N., Araujo F.M.C., Queiroz M.G.L., Cruz A.C.R., Vasconcelos B.H.B., Chiang J.O. (2018). In Situ Immune Response and Mechanisms of Cell Damage in Central Nervous System of Fatal Cases Microcephaly by Zika Virus. Sci. Rep..

[21] Oliveira D.N., Lima E.O., Melo C.F.O.R., Delafiori J., Guerreiro T.M., Rodrigues R.G.M., Morishita K.N., Silveira C., Muraro S.P., de Souza G.F. (2019). Inflammation Markers in the Saliva of Infants Born from Zika-Infected Mothers: Exploring Potential Mechanisms of Microcephaly during Fetal Development. Sci. Rep..

[22] Baer A., Kehn-Hall K. (2014). Viral Concentration Determination Through Plaque Assays: Using Traditional and Novel Overlay Systems. J. Vis. Exp..

[23] Sornjai W., Jaratsittisin J., Auewarakul P., Wikan N., Smith D.R. (2018). Analysis of Zika Virus Neutralizing Antibodies in Normal Healthy Thais. Sci. Rep..

[24] Quach A., Glowik S., Putty T., Ferrante A. (2019). Delayed Blood Processing Leads to Rapid Deterioration in the Measurement of the Neutrophil Respiratory Burst by the Dihydrorhodamine-123 Reduction Assay. Cytom. Part B Clin. Cytom..

[25] Miranda K.M., Espey M.G., Wink D.A. (2001). A Rapid, Simple Spectrophotometric Method for Simultaneous Detection of Nitrate and Nitrite. Nitric Oxide.

[26] Mulkey S.B., Arroyave-Wessel M., Peyton C., Bulas D.I., Fourzali Y., Jiang J., Russo S., McCarter R., Msall M.E., du Plessis A.J. (2020). Neurodevelopmental Abnormalities in Children with in Utero Zika Virus Exposure Without Congenital Zika Syndrome. JAMA Pediatr..

[27] Narasimhan H., Chudnovets A., Burd I., Pekosz A., Klein S.L. (2020). Animal Models of Congenital Zika Syndrome Provide Mechanistic Insight into Viral Pathogenesis during Pregnancy. PLoS Negl. Trop. Dis..

[28] Cugola F.R., Fernandes I.R., Russo F.B., Freitas B.C., Dias J.L.M., Guimarães K.P., Benazzato C., Almeida N., Pignatari G.C., Romero S. (2016). The Brazilian Zika Virus Strain Causes Birth Defects in Experimental Models. Nature.

[29] Camacho-Zavala E., Santacruz-Tinoco C., Muñoz E., Chacón-Salinas R., Salazar-Sanchez M.I., Grajales C., González-Ibarra J., Borja-Aburto V.H., Jaenisch T., Gonzalez-Bonilla C.R. (2021). Pregnant Women Infected with Zika Virus Show Higher Viral Load and Immunoregulatory Cytokines Profile with CXCL10 Increase. Viruses.

[30] Ornelas A.M.M., Pezzuto P., Silveira P.P., Melo F.O., Ferreira T.A., Oliveira-Szejnfeld P.S., Leal J.I., Amorim M.M.R., Hamilton S., Rawlinson W.D. (2017). Immune Activation in Amniotic Fluid from Zika Virus-Associated Microcephaly. Ann. Neurol..

[31] Reynolds C.J., Watber P., Santos C.N.O., Ribeiro D.R., Alves J.C., Fonseca A.B.L., Bispo A.J.B., Porto R.L.S., Bokea K., de Jesus A.M.R. (2020). Strong CD4 T Cell Responses to Zika Virus Antigens in a Cohort of Dengue Virus Immune Mothers of Congenital Zika Virus Syndrome Infants. Front. Immunol..

[32] Nikitina E., Larionova I., Choinzonov E., Kzhyshkowska J. (2018). Monocytes and Macrophages as Viral Targets and Reservoirs. Int. J. Mol. Sci..

[33] Bar-Or D., Bar-Or R., Rael L.T., Brody E.N. (2015). Oxidative Stress in Severe Acute Illness. Redox Biol..

[34] Sies H. (2018). On the History of Oxidative Stress: Concept and Some Aspects of Current Development. Curr. Opin. Toxicol..

[35] Yang T.C., Lai C.C., Shiu S.L., Chuang P.H., Tzou B.C., Lin Y.Y., Tsai F.J., Lin C.W. (2010). Japanese Encephalitis Virus Down-Regulates Thioredoxin and Induces ROS-Mediated ASK1-ERK/P38 MAPK Activation in Human Promonocyte Cells. Microbes Infect..

[36] Zhao J., Fan Y.-C., Sun F.-K., Zhao Z.-H., Wang L.-Y., Hu L.-H., Yin Y.-P., Li T., Gao S., Wang K. (2013). Peripheral Type I Interferon Receptor Correlated with Oxidative Stress in Chronic Hepatitis B Virus Infection. J. Interf. Cytokine Res..

[37] Labbé K., Saleh M. (2008). Cell Death in the Host Response to Infection. Cell Death Differ..

[38] Seet R.C.S., Lee C.-Y.J., Lim E.C.H., Quek A.M.L., Yeo L.L.L., Huang S.-H., Halliwell B. (2009). Oxidative Damage in Dengue Fever. Free Radic. Biol. Med..

[39] Castro Orozco R., Pinzón Redondo H.S., Alvis Guzmán N. (2015). A Systematic Review of Observational Studies on Oxidative/Nitrosative Stress Involvement in Dengue Pathogenesis. Colomb. Med..

[40] Bhaskar A., Munshi M.H., Khan S.Z., Fatima S., Arya R., Jameel S., Singh A. (2015). Measuring Glutathione Redox Potential of HIV-1-Infected Macrophages. J. Biol. Chem..

[41] Gostner J.M., Becker K., Fuchs D., Sucher R. (2013). Redox Regulation of the Immune Response. Redox Rep..

[42] Puttabyatappa M., Banker M., Zeng L., Goodrich J.M., Domino S.E., Dolinoy D.C., Meeker J.D., Pennathur S., Song P.X.K., Padmanabhan V. (2020). Maternal Exposure to Environmental Disruptors and Sexually Dimorphic Changes in Maternal and Neonatal Oxidative Stress. J. Clin. Endocrinol. Metab..

[43] Stephens H.A.F., Rothman A.L. (2010). HLA and Other Gene Associations with Dengue Disease Severity. Dengue Virus.

[44] Shapouri-Moghaddam A., Mohammadian S., Vazini H., Taghadosi M., Esmaeili S.A., Mardani F., Seifi B., Mohammadi A., Afshari J.T., Sahebkar A. (2018). Macrophage Plasticity, Polarization, and Function in Health and Disease. J. Cell. Physiol..

[45] Orzalli M.H., Kagan J.C., Children B., Avenue L. (2018). Apoptosis and Necroptosis as Host Defense Strategies to Prevent Viral Infection. Trends Cell Biol..

[46] Ignarro L.J. (1990). Biosynthesis and Metabolism of Endothelium-Derived Nitric Oxide. Annu. Rev. Pharmacol. Toxicol..

[47] de Noronha L., Zanluca C., Azevedo M.L.V., Luz K.G., dos Santos C.N.D. (2016). Zika Virus Damages the Human Placental Barrier and Presents Marked Fetal Neurotropism. Mem. Inst. Oswaldo Cruz.

[48] Glasauer A., Chandel N.S. (2013). ROS. Curr. Biol..

[49] Valero N., Mosquera J., Añez G., Levy A., Marcucci R., de Mon M.A. (2013). Differential Oxidative Stress Induced by Dengue Virus in Monocytes from Human Neonates, Adult and Elderly Individuals. PLoS ONE.

[50] Ivanov I.I., Zhou L., Littman D.R. (2007). Transcriptional Regulation of Th17 Cell Differentiation. Semin. Immunol..

[51] Olagnier D., Amatore D., Castiello L., Ferrari M., Palermo E., Diamond M.S., Palamara A.T., Hiscott J. (2016). Dengue Virus Immunopathogenesis: Lessons Applicable to the Emergence of Zika Virus. J. Mol. Biol..

[52] Gilbert-Jaramillo J., Garcez P., James W., Molnár Z., Clarke K. (2019). The Potential Contribution of Impaired Brain Glucose Metabolism to Congenital Zika Syndrome. J. Anat..

